# The Treatment of Consumption by Climate

**Published:** 1901-07-27

**Authors:** 


### The Treatment of Consumption by Climate.

Dr. C. Theodore Williams, F.R.C.P., in an opening address pointed out that climate has in all ages been considered to exercise an important influence in the treatment of pulmonary tuberculosis, though opinions have differed as to the kind of climate which is most beneficial. The principal climates utilised for the treatment of pulmonary tuberculosis may be grouped as: marine, dry warm climates, and mountain climates.

The temperate marine climates of the British Isles have been shown to be suitable for a large number of chronic cases, even when accompanied by pyrexia, and the avoidance of long voyages and the certainty of abundant and good food and home comforts are placed among their recommendations. The warm marine climates of Madeira, the Canaries, and the West Indies are beneficial in catarrhal tuberculosis, but not in phthisis generally. Sea voyages are probably the most successful form of marine climates, but this has to be said with many reservations as to the accommodation on board ship.

Dry warm climates include that of the Desert (especially the Egyptian) and those of the Mediterranean Basin. The Desert climate is remarkable for its dryness, sunshine, purity, and asepticity, and is especially adapted to open-air treatment. Owing to radiation the extremes of temperature are often great. General improvement in consumptives is perhaps more striking than local, but diminution in the amount of lung secretion and reduction of cough usually takes place. The Desert climate appears to act more decidedly in preventing the spread of tuberculous disease than in arresting that already present. It seems to most benefit cases of chronic cavity, especially in elderly persons and patients who are incapable of much exercise. The climate of the Riviera can be recommended in chronic phthisis, especially when complicated with inflammatory attacks, in strumous and laryngeal phthisis, and where there is rather extensive tuberculisation of the lungs in patients who from feeble circulation, shortwindedness, or advancing age are unable to bear the effects of a high altitude.

Mountain or high altitude climates vary according to latitude, but are all characterised by (1) diathermancy, or the increased facility with which the sun's rays are transmitted through the attenuated air, which causes a difference between sun and shade temperatures of 1° Fahr. for every rise of 235 feet (Denison); (2) by their asepticity, as shown by the preservation of meat for long periods; (3) by their physiological effects on the human body, as shown by the tanning of the skin, increase of both pulse and respiration rate, followed after a certain period of residence by a slowing and a deepening of the latter, and an extension of the thorax, also by an increase in the amount of urea and water excreted by the kidneys; more oxygen is absorbed and more carbonic acid expired by the lungs. The effect on cases of early tuberculous consolidation is remarkable, local pulmonary emphysema is produced around the tuberculous nodules, and the healthy portions of the lung become hypertrophied, necessitating enlargement of the thorax which can be proved in several ways. Expansion of the chest takes place unless opposed by extensive lung fibrosis or pleuritic adhesions.

The high altitudes have produced the best climatic results of all in the treatment of phthisis, but they are most successful in cases of early tuberculous consolidation, where they produce complete arrest in by far the larger proportion of patients. Also in hemorrhagic phthisis; but they are not equally successful in cases of excavation.

This, however, is to be noted not only in regard to high altitudes, but in regard to climatic treatment generally, namely, that it should be carried out under as strict medical supervision as sanatorium treatment.

